# The Complementary Role of Cardiopulmonary Exercise Testing in Coronary Artery Disease: From Early Diagnosis to Tailored Management

**DOI:** 10.3390/jcdd11110357

**Published:** 2024-11-05

**Authors:** Simone Pasquale Crispino, Andrea Segreti, Martina Ciancio, Dajana Polito, Emiliano Guerra, Giuseppe Di Gioia, Gian Paolo Ussia, Francesco Grigioni

**Affiliations:** 1 Department of Cardiovascular Sciences, Fondazione Policlinico Universitario Campus Bio-Medico di Roma, 00128 Rome, Italy; simone.crispino@unicampus.it (S.P.C.);; 2 Department of Movement, Human and Health Sciences, University of Rome “Foro Italico”, 00135 Rome, Italy; 3 Cardiology Division, Department of Biomedical, Metabolic and Neural Sciences, University of Modena and Reggio Emilia, Policlinico di Modena, 41125 Modena, Italy; 4 Institute of Sports Medicine and Science, National Italian Olympic Committee, 00135 Rome, Italy

**Keywords:** cardiopulmonary exercise testing, coronary artery disease, ischemia, functional capacity, risk stratification, prognostic value, exercise testing, stress test, chronic coronary syndrome

## Abstract

Coronary artery disease (CAD) remains a leading cause of morbidity and mortality worldwide, accounting for over 9 million deaths annually. The prevalence of CAD continues to rise, driven by ageing and the increasing prevalence of risk factors such as hypertension, diabetes, and obesity. Current clinical guidelines emphasize the importance of functional tests in the diagnostic pathway, particularly for assessing the presence and severity of ischemia. While recommended tests are valuable, they may not fully capture the complex physiological responses to exercise or provide the necessary detail to tailor personalized treatment plans. Cardiopulmonary exercise testing (CPET) offers a comprehensive assessment of the cardiovascular, pulmonary, and muscular systems under stress, potentially addressing these gaps and providing a more precise understanding of CAD, particularly in settings where traditional diagnostics may be insufficient. By enabling more personalized and precise treatment strategies, CPET could play a central role in the future of CAD management. This narrative review examines the current evidence supporting the use of CPET in CAD diagnosis and management and explores the potential for integrating CPET into existing clinical guidelines, considering its diagnostic and prognostic capabilities, cost-effectiveness, and the challenges associated with its adoption.

## 1. Background

Despite the continuous implementation of novel medical and interventional strategies for prevention and treatment, coronary artery disease (CAD) remains a leading cause of morbidity and mortality worldwide, underscoring the unmet need to improve patient outcomes [1]. It is estimated that CAD affects approximately 126 million individuals worldwide, representing about 1.72% of the global population [2,3]. The morbidity associated with CAD is considerable, as it is a leading cause of disability due to its impact on physical function and quality of life. Mortality rates also remain high, with CAD responsible for over 9 million deaths annually, making it the leading cause of death globally [2,3]. As CAD prevalence continues to rise, addressing these gaps becomes imperative for improving diagnostic precision and implementing timely interventions to mitigate disease progression and reduce cardiovascular events.

Regarding diagnostic approaches, historically, electrocardiogram (ECG) stress testing was considered the first-line tool for assessing CAD. However, its use has significantly declined, and it is now underutilized and less frequently recommended in contemporary clinical practice. Current guidelines still do not prioritize exercise testing in the early stages of CAD diagnosis, instead focusing on its use in selected patients to assess exercise tolerance, symptoms, arrhythmias, blood pressure response, and event risk (Class of recommendation I, level of evidence C) [4]. Furthermore, an exercise ECG may only be considered as an alternative for ruling in or out CAD when non-invasive imaging tests are unavailable, indicating a secondary role in the diagnostic process (Class of recommendation IIb, level of evidence B). The 2024 ESC guidelines emphasize the use of anatomic tests like coronary computed tomography (CCT) or functional tests such as single photon emission computed tomography (SPECT), cardiac magnetic resonance (CMR), or echocardiography with stress over exercise testing for the initial assessment of CAD.

Despite the shift toward these advanced imaging modalities, a gap still remains in the comprehensive assessment of CAD, particularly in understanding the interplay between cardiac, pulmonary, and muscular systems during physical exertion. Cardiopulmonary exercise testing (CPET), although not traditionally recommended in clinical guidelines for CAD management, offers a comprehensive alternative by continuously measuring gas exchange: specifically, oxygen uptake (VO_2_) and carbon dioxide production (VCO_2_), alongside electrocardiographic monitoring, blood pressure assessment, and ventilatory parameters analysis [5,6,7]. Moreover, the incremental diagnostic utility of CPET rests in the ability to obtain key variables that serve as surrogates for cardiac output (CO) and stroke volume (SV), enabling clinical evaluation in patients with suspected or confirmed CAD. In this setting, several studies have highlighted the potential of CPET as a valuable tool in the diagnosis and management of CAD [5,8,9].

Thus, a comprehensive approach that includes CPET may allow for a more detailed characterization of an individual’s exercise capacity and the correct identification of pathophysiological abnormalities that may not be apparent at rest in the context of CAD [7].

## 2. Methods

This narrative review involves a comprehensive search of databases like PubMed, Scopus, and Web of Science for relevant studies up to August 2024. We conducted a comprehensive review of the literature, including a thorough analysis of existing reviews, editorials, and clinical guidelines documents, to ensure a broad understanding of the subject matter. Furthermore, we specifically found and focused on 25 relevant studies that evaluated CPET’s diagnostic, prognostic, and therapeutic roles in the detection of myocardial ischemia and coronary artery disease, with comparisons to traditional methods, which are discussed throughout the present review. The aim is to provide an updated, integrative, accessible resource highlighting CPET’s potential in CAD diagnosis and management.

## 3. Discussion

### 3.1. Pathophysiological Mechanisms of Ischemia and Its Interaction with Gas Exchange

Ischemia, characterized by insufficient blood supply to tissues leading to inadequate oxygenation, significantly impacts the cardiovascular system, particularly during physical exertion [10]. The myocardium, highly dependent on a continuous oxygen supply, is especially vulnerable. When oxygen delivery is compromised, the heart shifts from aerobic to anaerobic metabolism, resulting in lactic acid accumulation and a decrease in pH, further impairing myocardial contractility and overall cardiac function. This metabolic disturbance reduces ATP production, hampering energy-dependent processes such as myocardial contraction, thus exacerbating the heart’s inability to meet the body’s oxygen demands during exercise [10,11].

From a hemodynamic perspective, ischemia induces several critical changes that further impair oxygen delivery and the removal of metabolic byproducts. One of the key consequences of ischemia is a reduction in SV, which leads to a decrease in CO [11]. This reduction in CO compromises the delivery of oxygenated blood to tissues, especially during periods of increased demand, such as physical exertion. To compensate for this reduction in SV, the body attempts to maintain CO by increasing the heart rate (HR). However, this compensatory mechanism is limited, especially as ischemia progresses. The heart’s ability to increase its rate diminishes due to impaired electrical conduction and reduced energy stores within the myocardium, ultimately leading to inadequate CO during peak exercise. Clinically, this manifests as exercise intolerance and early onset of fatigue.

Ischemia also triggers a sympathetic nervous system response, leading to vasoconstriction and an increase in systemic vascular resistance (SVR) [11,12]. This response aims to maintain blood pressure despite reduced CO, but the increased afterload—defined as the resistance against which the heart must pump—places additional strain on the ischemic myocardium. This further exacerbates the reduction in SV and CO. The coronary arteries, responsible for supplying blood to the heart muscle itself, are particularly vulnerable during ischemia [7]. Under normal conditions, coronary blood flow increases during exertion to meet the heightened metabolic demands of the myocardium. However, in the presence of significant atherosclerotic narrowing, this compensatory mechanism becomes insufficient, leading to myocardial ischemia and the associated clinical symptoms.

CPET is an advanced tool for assessing the physiological impact of ischemia, providing comprehensive insights into the integrated function of the cardiovascular, pulmonary, and muscular systems during exercise [13,14]. One of the most important CPET parameters is peak VO_2_, which represents the maximum amount of oxygen consumed during intense exercise [11]. Peak VO_2_ is a direct indicator of cardiorespiratory fitness and reflects the heart’s ability to deliver oxygenated blood to working muscles and the efficiency of those muscles in utilizing oxygen. In ischemic conditions, peak VO_2_ is typically reduced due to impaired CO and compromised oxygen delivery to tissues, making it a vital marker of the severity of ischemic heart disease and overall cardiovascular health. Another crucial parameter in CPET is the anaerobic threshold (AT), the point during exercise at which anaerobic metabolism begins to supplement aerobic metabolism due to insufficient oxygen delivery [11]. In ischemic patients, the AT occurs at a lower workload, indicating that the cardiovascular system cannot meet the metabolic demands of exercise, leading to an early reliance on anaerobic pathways and a quicker onset of fatigue. Oxygen pulse (O_2_ pulse), calculated as VO_2_ divided by HR, measures the efficiency of oxygen utilization and serves as an indirect indicator of SV during exercise [11]. In ischemic conditions, O_2_ pulse is reduced due to diminished SV, reflecting impaired cardiac function. A flattening of or decrease in the O_2_ pulse trajectory during exercise is often indicative of myocardial ischemia.

Ventilatory efficiency, assessed by the V_E_/VCO_2_ slope, measures the efficiency of the lungs in eliminating carbon dioxide (CO_2_) [11]. A steeper slope indicates poor ventilatory efficiency, commonly observed in heart failure and ischemic heart disease. This inefficiency is often due to increased dead space ventilation or ventilation–perfusion mismatch, both of which are exacerbated by the hemodynamic changes associated with ischemia. Additionally, heart rate response (HRR) during exercise, including the recovery phase, provides insights into autonomic function and cardiovascular health [5,11,12]. A blunted HR response, known as chronotropic incompetence, is common in ischemic heart disease and is associated with poor outcomes. The chronotropic index, which compares the actual HR increase during exercise to the expected increase, is often reduced in ischemic patients, indicating an impaired ability to meet the demands of exercise [11,12].

Thus, the behavior of these parameters reflects the underlying pathophysiological mechanisms driving these changes, primarily related to the imbalance between oxygen supply and demand. As myocardial oxygen demand increases significantly during physical exertion to support enhanced cardiac output, the limited supply cannot meet this demand due to restricted coronary blood flow. Consequently, the heart attempts to compensate by increasing HR and contractility, but these compensatory mechanisms are insufficient in the presence of significant ischemia, leading to further deterioration in cardiac function and reduced exercise capacity, as observed during CPET.

### 3.2. Limitations of Traditional Ergometric Testing and the Advantages of Cardiopulmonary Exercise Testing

Historically, ECG monitoring during exercise has been the most popular non-invasive test for investigating coronary artery disease (CAD). It is simple to perform, safe, and cost-effective. During the test, the patient exercises using a treadmill or bicycle while ECG monitoring is conducted. The sensitivity (true-positive rate) of an exercise ECG is 45–50%, and its specificity (true-negative rate) is about 85–90%, making it more reliable for excluding CAD rather than confirming it [15].

Exercise testing provides valuable clinical information, such as establishing exercise tolerance, assessing symptoms, and evaluating blood pressure response during exertion. It can also be helpful in monitoring symptomatology in patients undergoing medical therapy. However, the 2024 ESC guidelines on chronic coronary syndromes, similarly to the 2019 edition, recommend an exercise ECG only when non-invasive imaging is unavailable for ruling out CAD [4,16]. In this case, a key indicator to stratify mortality risk in patients with suspected or newly diagnosed CAD is using the Duke treadmill score [16,17].

The AHA/ACC Guidelines for the management of patients with chronic coronary disease confirm the use of exercise testing in symptomatic patients with an intermediate risk of acute coronary syndrome [3]. These guidelines also endorse its use for predicting cardiovascular events, assessing chronotropic competence, exercise-induced symptoms, and unexplained syncope in patients at intermediate or high risk of CAD, and evaluating response after medical or surgical interventions in patients with valvular disease, arrhythmias, or other cardiac pathologies.

While ECG monitoring during exercise has traditionally been a popular non-invasive method for investigating CAD, it does present notable limitations. Specifically, it has no diagnostic value in patients with ECG abnormalities that prevent the interpretation of ST-segment changes during exercise, such as left bundle branch block, accelerated rhythms, Wolff–Parkinson–White syndrome, and ST-segment depression (greater than 0.1 mV on resting ECG), or in patients being treated with digitalis, as will be discussed throughout the text [15]. Anti-ischemic drugs that lower HR and myocardial workload can also lead to false negative results. Furthermore, traditional exercise testing is less useful than exercise imaging-based methods for localizing ischemia, and has a lower diagnostic value in women [15].

Traditional exercise testing mainly correlates ischemic cascades with ECG changes, confirming positive or negative results based on ST-segment depression. However, other factors; such as the increase in R-wave amplitude during exercise; the prolongation of P-wave duration; the relationship between ST-segment depression and HR, the blood pressure, and HRR to exercise; and the occurrence of chest pain, heart sounds, and palpitations; are non-specific and often less reliable [18].

Moreover, ECG changes occur late in the course of systolic dysfunction, which contributes to the test’s low sensitivity. ST-segment depression reflects only a functional sign of inadequate oxygen supply to meet myocardial demands and can be caused by various conditions, such as anemia or left ventricular hypertrophy, which diminishes the specificity of exercise testing [15,19].

In the past two decades, CPET has emerged as a viable approach for assessing exercise-induced ischemia, addressing some of the limitations of traditional exercise testing. CPET allows for real-time measurement of various parameters, enabling the detection of ischemia-induced changes in response to increasing work rates [12]. A landmark study by Belardinelli et al. demonstrated that CPET improves diagnostic accuracy compared to stress ECG, significantly enhancing sensitivity and specificity in patients with documented or suspected macrovascular CAD [5].

CPET offers incremental diagnostic utility by measuring key variables that function as surrogates for CO and SV, enabling clinical evaluation in patients with suspected or confirmed CAD.

Furthermore, a comprehensive assessment of aerobic capacity, ventilatory efficiency, and metabolic responses during exercise is possible, which surpasses the capabilities of the traditional exercise ECG. Parameters such as VO_2_, the V_E_/VCO_2_ slope, and oxygen pulse are particularly useful in detecting subtle cardiopulmonary abnormalities and ischemia-induced changes in response to increased workload, thus improving the sensitivity and specificity of CAD diagnosis.

CPET has also been demonstrated to be superior in diagnosing CAD in patients with non-diagnostic or inconclusive results from traditional exercise tests [10]. When baseline ECG abnormalities prevent accurate interpretation, CPET compensates by providing additional data on the cardiopulmonary system’s response to exercise, which is not reliant solely on ECG changes [20]. Furthermore, it is less influenced by medications, such as beta-blockers, which can mask ischemic changes during traditional exercise tests. This allows for more accurate assessment of exercise capacity and ischemic threshold, particularly in cases of iatrogenic chronotropic incompetence, where patients cannot achieve the necessary HR targets during conventional ergometric tests. In CPET, the threshold of exercise maximality is determined by the respiratory exchange ratio (RER), which is considered maximal when RER exceeds 1.05 in patients with chronic cardiac or pulmonary diseases, or 1.10 in the general population [21,22].

From a prognostic and follow-up perspective, CPET provides additional prognostic value through parameters like peak VO_2_ and the V_E_/VCO_2_ slope, which are strong predictors of cardiovascular outcomes, offering more detailed insights than the Duke Treadmill Score [20]. CPET is particularly beneficial for women, athletes, and heart failure patients, as it provides accurate differentiation between physiological and pathological responses, detects subtle ischemic changes, and assesses ventilatory efficiency.

**Figure 1:**
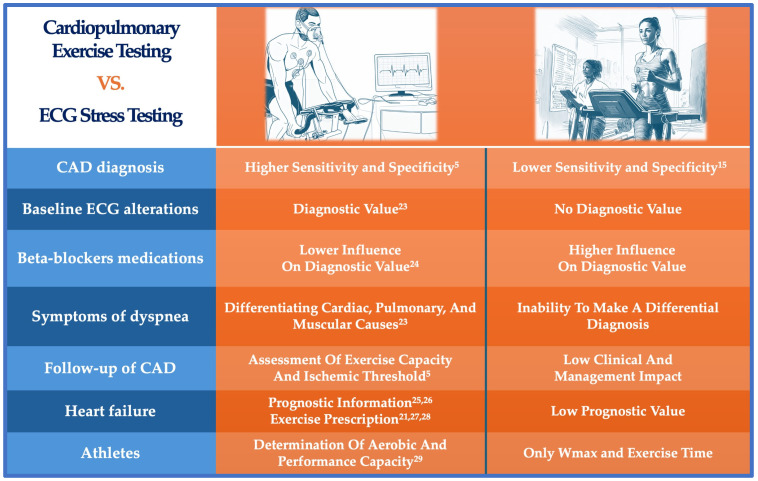
Central illustration. Differences between cardiopulmonary exercise testing and classic ergometric test. CPET demonstrates higher sensitivity and specificity for diagnosing coronary artery disease (CAD), even when baseline ECG alterations are present, whereas ECG stress testing has lower diagnostic accuracy and is ineffective in such cases [5,15,23]. Beta-blocker medications minimally impact CPET’s diagnostic value, unlike ECG stress testing, which is more influenced by these drugs, especially in heart failure patients [24]. CPET is superior for differentiating cardiac, pulmonary, and muscular causes of dyspnea [23] and provides valuable data for assessing CAD follow-up [5] and heart failure prognosis [25,26], guiding exercise prescriptions [21,27,28]. In contrast, ECG stress testing contributes less to clinical management and has limited prognostic value. For athletes, CPET assesses full aerobic and performance capacity, while ECG stress testing only measures maximal workload (Wmax) and exercise time [29]. Overall, CPET offers greater diagnostic and prognostic capabilities compared to ECG stress testing.

The main findings discussed here are summarized in Figure 1 (Central Illustration).

### 3.3. The Evolution of CPET in the Assessment of Myocardial Ischemia: Not Only VO_2_

Early investigation and advancements in VO_2_. Early investigations demonstrated significant advancements in the use of CPET for assessing myocardial ischemia beyond traditional VO_2_ measurements. In 2002, a foundational study by Belardinelli et al. [21] assessed the diagnostic accuracy of CPET in comparison to traditional stress ECG, using SPECT myocardial scintigraphy as the reference standard. This study introduced a two-variable model based on O_2_ pulse flattening duration and the ΔVO_2_/ΔWR slope, which achieved markedly higher sensitivity (87%) and specificity (74%) for detecting exercise-induced myocardial ischemia (EIMI) than stress ECG (sensitivity 46%, specificity 66%) in a cohort of 202 patients with documented CAD. These findings underscored CPET’s enhanced capability for ischemia detection, especially in patients with a significant ischemic burden.

In related research, Bussotti et al. [30] reported that patients with epicardial disease displayed significantly reduced peak VO_2_ (68%) and a flattened ΔVO_2_/ΔWR slope post-anaerobic threshold (AT) compared to patients without epicardial disease and to healthy controls. Additionally, Belardinelli et al. [8] observed that patients achieving a peak VO_2_ above 91% of the predicted value and lacking ischemia-related VO_2_ alterations had a 100% negative predictive value for obstructive coronary artery disease (O-CAD), suggesting CPET’s substantial potential as a reliable diagnostic tool in clinical practice.

Li et al. [31] further elaborated on these findings, reporting a proportional decrease in mean peak VO_2_ with increasing O-CAD burden, thus establishing a gradient from healthy individuals to those with severe O-CAD. Declines in both peak stroke volume (SV) and peak heart rate (HR) were also observed with the progression of atherosclerosis, supporting the utility of CPET in identifying the physiological impact of CAD.

An emerging focus in CPET research involves assessing parameters during the recovery phase of exercise. Popovic et al. [14] explored gas exchange metrics during CPET recovery to predict CAD severity and prognosis in a small cohort of 40 patients (79% male) using both treadmill and recumbent ergometry. The study found that recovery-phase changes in carbon dioxide output (ΔVCO_2_) and oxygen uptake (ΔVO_2_) recovery-to-peak ratios effectively differentiated patients with one- or two-vessel coronary artery stenosis from those with three-vessel stenosis. Receiver operating characteristic (ROC) analysis indicated that treadmill testing offered higher predictive value for CAD severity than recumbent cycle ergometry. These findings suggest that CPET recovery-phase gas exchange may serve as a valuable indicator of CAD severity, as impaired pulmonary gas exchange during recovery correlates with a greater ischemic burden.

Finally, Yoshida et al. [32] identified post-ischemic threshold heart rate increases as a key contributor to cardiac output and peak VO_2_. The study highlighted that beta-blockers could induce exercise intolerance by suppressing compensatory heart rate responses beyond the ischemic threshold, thus impairing exercise tolerance in patients with angina. This research provides important insights into the reliance of cardiac output on HR and SV during exercise, particularly under pharmacological intervention.

Oxygen pulse. For what concerns the O_2_ pulse, a reliable measure of cardiac performance during exercise, Munhoz et al. [33] initially explored the relationship between the O_2_ pulse response and inducible ischemia, as detected by nuclear stress imaging, particularly using SPECT. The study found that only patients with extensive ischemia on the SPECT nuclear study had significantly reduced peak O_2_ pulse and peak VO_2_. This research demonstrated that reduced peak SV and CO were linked with high ischemic burdens. However, subsequent findings by Mazaheri et al. [34] showed that the duration of the O_2_ pulse plateau was similar between the OCAD and non-OCAD groups, and the reduction in peak VO_2_ observed in the OCAD group was not statistically significant. Nevertheless, this study highlighted the potential of the ventilatory equivalent for carbon dioxide (VE/VCO_2_) as a predictive marker for CAD when its value exceeds 35. Petek et al. [35] observed that a plateau in the O_2_ pulse was not a useful predictor of OCAD, suggesting that the O_2_ pulse parameter should be integrated with other CPET metrics rather than used in isolation.

Finally, Ganesananthan et al. [36] revealed that patients who exhibited an O_2_ pulse plateau during CPET had higher ischemic DSE scores and lower fractional flow reserve (FFR) compared to those without an O_2_ pulse plateau.

Efficiency of oxygen delivery and consumption. Tajima et al. [37] demonstrated that patients with CAD exhibit a significant decrease in the ΔVO_2_/ΔWR slope above the VAT and IT, along with delayed VO_2_ recovery kinetics compared to those without CAD. The use of coronary angiography as a comparative tool validated the CPET findings, confirming that a higher CAD burden is associated with slower increases in cardiac output post-VAT and a prolonged return to baseline due to the ischemic burden. This study highlighted the value of CPET in assessing cardiac output dynamics and further provided a clear link between CPET and traditional imaging modalities like coronary angiography. Similar results in terms of reduced ΔVO_2_/ΔWR slope were subsequently obtained by a study of Uliari et al. [38].

Van de Sande et al. [39] showed that athletes with abnormal stress ECGs presented an attenuated O_2_ pulse slope, a decreased ΔVO_2_/ΔWR ratio, and an increased heart rate–work rate (ΔHR-WR) slope after the AT, consistent with microvascular ischemia.

Chaudhry et al. [20] demonstrated that heart rate acceleration as a function of work rate (WR) after the AT improved the diagnostic accuracy in detecting exercise-induced myocardial ischemia (EIMI) compared to traditional stress ECG.

Coronary microcirculation. Also of note, considering the importance of coronary microvascular circulation given in the latest ESC guidelines on chronic coronary syndromes, a correlation between coronary microvascular dysfunction (CMD) and peak VO_2_ exists and was demonstrated in a study by Bechsgaard et al. [40], which showed that peak VO_2_ was significantly reduced (17.3 vs. 27.3 mL/kg/min) in patients with CMD, regardless of other cardiovascular risk factors.

Prognostic power after revascularization for myocardial infarction. For what concerns findings of CPET and their prognostic power on patients with a recent acute coronary syndrome (ACS) treated with percutaneous coronary intervention (PCI), Niu et al. [41] conducted a study on 184 patients, identifying four CPET variables—premature termination, low peak VO_2_, HRR, and V_E_/VCO_2_ slope—as predictors of cardiovascular events after PCI. Furthermore, reduced peak cardiac output (as indicated by low peak VO_2_) and chronotropic incompetence were established as parameters of poor prognosis in CAD patients.

In addition, Smarz et al. [42] explored the determinants of exercise capacity in 81 patients treated for acute myocardial infarction (AMI), with a mean age of 58 years and presenting 70% males. The study combined CPET with stress echocardiography (CPET-SE) and found that exercise capacity in patients with left ventricular ejection fraction (LVEF) greater than 40% was related to peak HR and peripheral oxygen extraction, but not to peak SV. Patients showing exercise-induced myocardial ischemia (EIMI) on CPET-SE were excluded from the final analysis. The study emphasized that the semi-recumbent position required for stress echocardiography might limit preload, thereby affecting SV and influencing the results. This research highlighted the importance of the integration and complementarity of CPET with other modalities to fully understand exercise capacity limitations in post-AMI patients.

Subclinical changes in at-risk patients. Furthermore, Gulsin et al. [15] assessed the relationship between subclinical cardiac dysfunction and peak VO_2_. The study found that subjects with Type 2 diabetes (T2D) had increased concentric left ventricular remodeling, reduced myocardial perfusion reserve (MPR), and markedly lower aerobic exercise capacity (peak VO_2_ 18.0 vs. 27.8 mL/kg/min) compared to controls. Only MPR and left ventricular diastolic filling pressure were independently associated with peak VO_2_ in subjects with T2D, indicating that microvascular dysfunction and diastolic dysfunction are key drivers of reduced exercise capacity in this population.

Supplementary Table S1 provides a resume of the main studies on the use of CPET in the assessment of myocardial ischemia.

### 3.4. CPET Parameters in the Assessment of CAD: A Practical Approach

CPET provides a multisystemic and integrated approach to identifying inducible ischemia, making it useful in the early diagnosis of CAD. Its accuracy stems from the ability to assess the increase in cardiac output and SV during physical stress, while also analyzing respiratory and metabolic parameters. As workload increases, myocardial oxygen demand rises, requiring increased chronotropic activity and arteriolar vasodilation, which are facilitated by the regeneration of high-energy phosphate compounds like adenosine triphosphate (ATP). However, in atherosclerotic disease of the coronary arteries, the obstruction to blood flow, combined with reduced diastolic time (exacerbated by an excessive chronotropic response), compromises adequate perfusion, leading to ischemia. CPET provides early and additional parametric information in case of inducible ischemia, compared to traditional exercise stress testing, by identifying mechanical dysfunctions that occur before electrocardiographic changes or symptom onset. Thus, CPET serves both diagnostic and prognostic purposes through the analysis of these parameter alterations.

**Figure 2:**
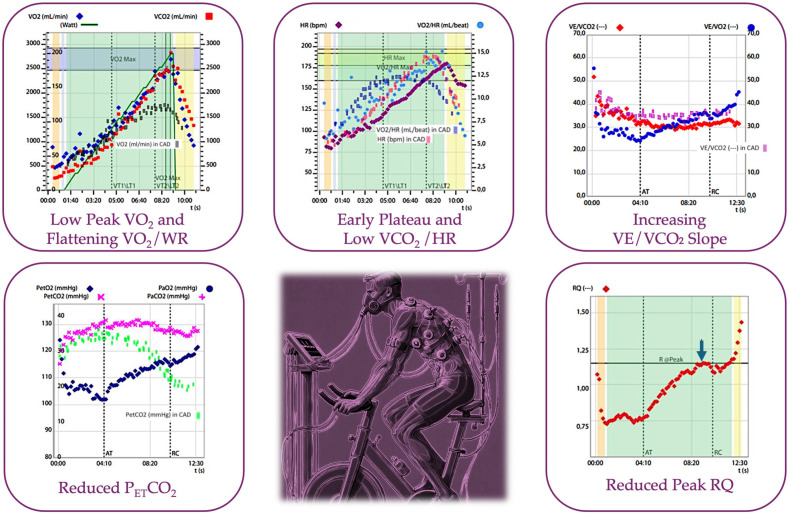
Changes in gas exchange parameters in the setting of detectable myocardial ischemia.

Key changes are depicted in Table 1 and Figure 2.

VO_2_ peak. This is a measure of peak exercise oxygen consumption and serves as an important indicator of cardiorespiratory fitness. According to Fick’s law, peak VO_2_ reflects the body’s capacity to increase CO at peak exercise, providing insight into both systolic and diastolic function. It is one of the most significant prognostic predictors in patients with heart disease, as its value is inversely correlated with the risk of myocardial infarction and heart failure. Several studies have emphasized the diagnostic value of peak VO_2_, consistently showing that patients with CAD exhibit significantly lower VO_2_ peak values. Similar trends are observed for other related parameters, such as VO_2_ measured at the AT and VO_2_/kg [14,43,44,45].

Specifically, Skalski et al. [46] demonstrated that peak VO_2_ in CAD patients reached only two-thirds of the predicted value, highlighting a substantial limitation in functional capacity. Additionally, multiple studies have shown that a lower peak VO_2_ value is associated with an increased risk of adverse cardiovascular events in patients with established coronary artery disease.

In the general population, normal VO_2_ peak values typically range from 20 to 40 mL/kg/min, depending on age, sex, and fitness level. A VO_2_ max below 20 mL/kg/min is generally considered abnormal and indicative of significant cardiopulmonary impairment [23]. Fujimoto et al. specifically found that VO_2_ peak values below 16 mL O_2_/kg/min were associated with an increased cardiovascular risk within two years following an acute myocardial infarction treated with percutaneous coronary intervention (PCI) [47,48,49,50].

Oxygen Uptake at AT. VO_2_ at the AT is another measure of aerobic exercise capacity. From a metabolic perspective, the achievement of the AT represents the point at which aerobic metabolism no longer meets metabolic demands, and anaerobic metabolism prevails. This typically occurs at 40–60% of VO_2_ max in healthy individuals. An earlier onset of AT suggests impaired cardiovascular or muscular function [23]. VO_2_ at AT reflects the efficiency of mitochondrial O_2_ utilization and provides insights into the severity of cardiac impairment and cardiac function. When earlier, it also assumes a prognostic role, as demonstrated by analysis of the MECKI score database [26].

Oxygen Pulse. The oxygen pulse is a direct estimate of SV, which can be derived from the Fick equation: VO_2_ = HR × SV × ΔO_2_(a-v). Assuming ΔO_2_(a-v) remains constant, the oxygen pulse (VO_2_/HR) equals SV [22]. Therefore, based on previous discussion, it serves as an indicator of an adequate cardiac response to exercise. Under physiological conditions, the increased oxygen demand during exercise is met by an increase in cardiac output, determined by the variables HR and SV. In healthy individuals, there is an adequate response characterized by an increase in SV and a reduced HR at the same workload. In contrast, patients with heart disease exhibit an inadequate increase in SV, leading to an increase in chronotropic activity (HR). This results in cardiac inefficiency. In cases of cardiogenic limitation, the expected curve will reach an early plateau and settle at lower values than anticipated. A recent study showed that patients with CAD undergoing CPET had lower VO_2_/HR values than the healthy population, with a specificity of 92.4% [6].

Furthermore, a recent study has demonstrated an association between cardiovascular risk factors and altered oxygen pulse (VO_2_/HR) in asymptomatic men aged 20–60 years with normal cardiorespiratory fitness (CRF) and peak VO_2_ [51].

A study by Chaudhry et al. showed increased specificity and sensitivity in identifying patients with CAD. The study analyzed oxygen pulse variables and early HR increases in relation to exercise exertion. It demonstrated that the reduction in oxygen pulse was directly proportional to the degree of coronary obstruction, the severity of ischemic disease, and the symptom burden [20].

VO_2_/WR. In patients who develop cardiac ischemia during exercise, resulting in a sudden drop in cardiac output, oxygen supply is compromised, and anaerobic metabolism significantly contributes to ATP generation. This leads to a relative flattening of VO_2_/WR (work rate), which serves as a useful marker for suspecting coronary ischemia. Studies have shown that in subjects with coronary ischemia, the flattening of VO_2_/WR usually correlates with the flattening of the oxygen pulse, indicating the onset of ischemic myocardial dysfunction. In healthy subjects, normal oxygen uptake is 10 mL/min/W, and this increase remains a linear function during exercise [19]. Belardinelli et al. identified a slope cut-off of <3.9 mL/min/W to detect the onset of ischemia [5].

ΔHR-WR Slope. Chaudhry et al. [20,52] explained a pathophysiological mechanism through the ΔHR-WR slope parameter. To maintain peripheral perfusion to skeletal muscles during exercise, the sympathetic autonomic nervous system accelerates HR as a compensatory mechanism. This compensatory response in late exercise was quantified as the change in the HR slope parameter of working velocity by comparing the HR slope in the last 2 min of exercise with that at mid-exercise. The study aimed to improve diagnostic accuracy for non-obstructive CAD using CPET. The effectiveness of the ΔHR-WR slope in detecting under-treated atherosclerosis was compared to a traditional stress ECG. The study included 208 symptomatic patients without prior heart or lung disease, all of whom underwent coronary angiograms, and 116 healthy controls. Results showed that the ΔHR-WR slope significantly improved sensitivity for detecting both obstructive and non-obstructive CAD without compromising specificity. In men, the area under the receiver operating characteristic (ROC) curve increased from 60% to 94% for non-obstructive CAD and from 64% to 80% for obstructive CAD. In women, it increased from 64% to 85% for non-obstructive CAD and from 66% to 90% for obstructive CAD. The ΔHR-WR slope reclassified abnormal studies from 22% to 81% in the non-obstructive CAD group and from 18% to 84% in the obstructive CAD group. The study concluded that CPET, using the ΔHR-WR slope, is superior to the stress ECG for identifying cardiac dysfunction in symptomatic patients with non-obstructive CAD, offering a low-cost, safe, and widely available first-line diagnostic tool, particularly for women. Moreover, symptomatic patients with varying degrees of coronary plaque showed accelerated HR (positive ΔHR-WR slope; values > 15% are pathological) in late exercise [52].

V_E_/VCO_2_ Slope. Ventilatory efficiency, assessed by the V_E_/VCO_2_ slope, is an index of the ability to eliminate CO_2_ produced during exercise through ventilation. However, in CAD, the imbalance between O_2_ demand and supply leads to an early shift to anaerobic metabolism, increasing CO_2_ production, which acts as a ventilatory stimulus.

The reduced cardiac output, as seen in CAD, affects both right and left circulations, leading to increased left atrial pressure and pulmonary congestion. This impairs pulmonary perfusion and gas exchange at the capillary level, and consequently increases the V_E_/VCO_2_ slope. These mechanisms are driven by an imbalance between endothelial vasodilators (e.g., nitric oxide and prostacyclins) and the vasoconstrictor effects of catecholamines released during exercise.

Thirapatarapong et al. [53] demonstrated that patients with chronic obstructive pulmonary disease (COPD) and CAD had an altered V_E_/VCO_2_ slope compared to those with COPD, during CPET.

Several authors have highlighted a correlation between an increased VE/VCO_2_ slope and a higher incidence of adverse cardiovascular events, giving the VE/VCO_2_ slope a negative prognostic significance similar to peak VO_2_ [54]. A slope of less than 35 is considered normal, while higher values indicate ventilatory inefficiency, often associated with heart failure or pulmonary vascular disease [23].

Chronotropic Response. While both healthy individuals and those affected by CAD can achieve similar peak HR values, the HR trend during exercise offers more valuable information. The chronotropic index (HR-VO_2_ slope) reflects the extent to which changes in cardiac output in response to exercise depend on HR.

Under normal conditions, once the first threshold is reached, the HR slope remains constant or decreases. However, in patients with inducible myocardial ischemia, the HR slope increases abruptly once the first threshold or AT is crossed.

Additionally, an early increase in HR, even before reaching AT, is associated with cardiac disease and poor prognosis. A study involving asymptomatic athletes at low cardiovascular risk demonstrated high specificity in detecting microvascular dysfunction and inducible ischemia. This was primarily reflected in an early increase in HR after AT, as well as a reduced oxygen pulse and ΔVO_2_/WR [39].

Although not specific to CPET but related to exercise physiology in general, heart rate recovery (HRR) represents a significant parameter to assess in exercise testing. Cole et al. [55] defined HRR as the decrease in HR from peak exercise to one minute post-exercise. Other authors, including Shetler et al., extended the HR measurement interval from 30 s to 10 min [56].

During exercise, the increase in HR contributes to increased cardiac output, which is made possible by a rise in sympathetic activity and a decrease in vagal discharge. This dual mechanism increases both HR and SV, as well as myocardial contractility, to meet the energy demands of the exercising muscles.

The autonomic contribution to post-exercise cardiodeceleration, and thus the restoration of basal HR, is primarily determined by the cessation of the exercise stimulus from the brain (cerebral cortex—central command) followed by an increase in parasympathetic tone [19]. The rate at which HR returns to baseline and the time required to recover after moderate to intense exercise are commonly used indicators of autonomic nervous system function and cardiovascular performance during exercise [57].

A delayed decrease in HR during the first minute after exercise is an indicator of autonomic dysfunction and has been suggested as a powerful and independent predictor of increased risk of all-cause mortality.

In the CAD setting, Cole et al., through a six-year follow-up study of patients with established CAD, demonstrated that HRR is an important predictor of cardiovascular death [55].

Another study with a 7-year follow-up showed that low HRR values were predictive of a higher mortality risk, regardless of the severity of coronary artery disease [58].

In conclusion, HRR provides important diagnostic and prognostic information during exercise in patients with CAD, regardless of disease severity [59].

Exercise Time. Although not specific to CPET, exercise duration is a clear determinant in assessing functional capacity. It is well established that patients with coronary artery disease (CAD) and heart failure (HF) have reduced exercise tolerance, which is an important predictor of adverse cardiovascular events and mortality [60]. Exercise duration is one of the key parameters determining exercise tolerance. The American Heart Association (AHA) indicates exercise capacity is the strongest predictor of mortality risk, alongside established cardiovascular risk factors (including smoking, hypertension, Type 2 diabetes mellitus, and dyslipidemia) [61]. Several authors have hypothesized that the lower exercise tolerance levels observed in CAD patients are related to factors such as chronotropic incompetence, lung mechanics disorders, inspiratory muscle fatigue, ineffective gas exchange, and abnormal peripheral muscle metabolism, which contribute to a “fear of exercising” [62,63].

During a treadmill exercise stress test, the Duke Treadmill Score (DTS) is one of the most widely used and reliable indices for estimating the risk of CAD. It is a non-invasive and easy-to-use tool that combines exercise time, the Bruce protocol, maximum ST-segment deviation on ECG, and exercise-induced angina [17]. DTS has been validated for risk stratification in patients with CAD for future events, such as myocardial infarction or stroke [64,65].

Salokari et al. were the first to apply the DTS to cycle ergometer exercise testing, commonly preferred in European countries, and compared the diagnostic ability of the DTS with its individual components. Since exercise duration can also be expressed in terms of metabolic equivalents (METs), the study showed that DTS predicts cardiovascular mortality even during cycle ergometer testing, but METs alone were found to be a better predictor [66].

However, while exercise time is a significant value in a CPET performed on a treadmill, in the case of a ramp CPET with a cycle, exercise duration is not as significant unless a previous exercise test using the same ramp protocol is available for comparison.

P_ET_CO_2_. End-tidal carbon dioxide (P_ET_CO_2_) provides useful information about ventilation–perfusion (V_A_/Q) mismatch. Cardiac patients often experience inadequate cardiac output during exercise, which leads to hypoperfusion of skeletal muscles with an early shift to anaerobic metabolism. This results in lactic acid accumulation, tissue hypoxia, muscle fatigue, and dyspnea, even at low exercise intensities [67,68,69].

Additionally, the risk of pulmonary congestion, which can reduce lung compliance and induce rapid, shallow breathing, should also be considered [70]. This situation leads to an altered ventilation–perfusion ratio, increasing physiological dead space at the expense of tidal volume [28]. During CPET, this manifests as an increase in the ventilatory equivalent for CO_2_ elimination and a steeper minute ventilation slope as a function of CO_2_ production [27]. Matsumoto et al. [71] have shown that cardiac patients exhibited reduced P_ET_CO_2_ with a positive P(a-ET)CO_2_ gradient during exercise, suggesting ventilation–perfusion mismatch or increased alveolar dead space. This correlated with an inadequate increase in cardiac output and poor perfusion of ventilated alveoli during exercise.

**Table 1:** Main CPET parameters of importance in ischemia detection.

Value	Reference Value	Explanation
VO_2_ max [5]	>85% of the predicted	Reduced in ischemia due to impaired oxygen delivery from reduced cardiac output, limiting maximal aerobic capacity.
V_E_/VCO_2_ slope [54]	<35 L/min/L	Elevated in ischemia as inefficient ventilation and gas exchange occur due to poor perfusion.
Oxygen Pulse [54]	>80% of the predicted	Flattened or declining during exercise may indicate reduced stroke volume, often seen in myocardial ischemia.
P_ET_CO_2_ [72]	36–42 mmHg	Decreased in ischemia due to impaired perfusion, affecting CO_2_ clearance from the lungs.
Heart Rate Recovery [55]	<15 bpm	Delayed heart rate recovery post-exercise indicates autonomic dysfunction, which is often observed in myocardial ischemia.
Exercise Time [5]	8–12 min	Shorter exercise duration is common in ischemia due to early onset of symptoms like angina and reduced cardiac output limiting exercise.

### 3.5. Prognostic Value of CPET and Cardiorespiratory Fitness Versus Ergometric Test

Patients with suspected ischemic heart disease undergo non-invasive examinations to establish the presence of ischemic heart disease and define long-term prognosis (prognostic pathway). Prognostic stratification aims to identify patients who might benefit from revascularization or those who may be better suited for medical therapy.

To test prognostic accuracy in CAD, traditional stress tests, such as ergometric testing, are widely used and continue to hold a place in clinical guidelines, particularly in the prognostic setting.

Several stress-test variables are associated with prognosis. The most important is the duration of exercise. Other important variables include angina, the extent of ST-segment deflection, and various hemodynamic responses, such as blood pressure response and HR changes during exercise (e.g., chronotropic incompetence), and HR after exercise [73].

One of the most useful and widely cited scoring systems for predicting prognosis and guiding management in patients with suspected coronary artery disease is the Duke Treadmill Score (DTS). This considers three independent variables (exercise time, ST-segment deviation, and angina index) [17].

A Duke Treadmill Score > 5 indicates a low risk for cardiovascular events (with a predicted 4-year survival rate of 99%), and these patients do not need further investigation with coronary angiography. A score < –10 indicates a high risk for cardiovascular events (predicted 4-year survival of 79%), and these patients require further investigation with coronary angiography. Scores between 4 and –10 represent intermediate risk [17].

However, it has recently been observed that at least half of the patients undergoing exercise testing are unable to perform adequate treadmill exercise due to lower limb disabilities or the use of medications that act as exercise substitutes, making the Duke Treadmill Score less relevant. Additionally, the Duke Treadmill Score does not include variables such as heart rate recovery, which has been found to be highly predictive of mortality [74].

However, the outcomes presented so far are not CPET-specific. Compared to traditional cardiac function assessment tools, such as exercise electrocardiography, CPET has several prognostic advantages. It can measure a wider range of variables related to cardiorespiratory function, providing greater prognostic accuracy. VO_2_ peak is considered the gold standard for assessing cardiorespiratory fitness (CRF), and it is strongly associated with cardiovascular mortality.

Liu et al. conducted a study involving 280 coronary angiography patients undergoing CPET. Correlation analyses were performed between CRF and the severity of coronary artery disease. A correlation analysis was performed between CRF and the severity of CAD. Their study demonstrated a strong association between CRF and the severity of CAD. A combination of traditional clinical risk factors and CRF is valuable in predicting CAD severity. CRF screening via CPET provides a strategy for categorizing individuals into severe, mild, or moderate CAD [7].

Furthermore, a scientific update from the American Heart Association (AHA) [75] affirmed that CRF may provide additional prognostic value for CVD risk and associated mortality, in addition to traditional risk factors such as hypertension, smoking, obesity, hyperlipidemia, and Type 2 diabetes mellitus.

Despite the confirmed usefulness of CRF, it is not routinely included in many CVD management guidelines or used for risk stratification.

This may be due to the lack of widespread information regarding CRF’s role in predicting long-term mortality in adults with CVD. Ezzatvar et al. [76] conducted a study to quantify the association between CRF in adults with established CVD and the risk of all-cause mortality and CVD.

An analysis of 21 prospective studies concluded that better CRF is associated with a lower risk of all-cause mortality and CVD. This study supported the use of CRF as a powerful predictor of mortality in this population. Moreover, in patients with known CAD, CPEY can provide useful prognostic information regarding long-term mortality. Kavanagh et al. demonstrated that each 1 mL/kg/min increase in peak VO_2_ reduces mortality by 10% in women with known CAD [49].

CPET can also serve as an excellent prognostic marker for stable CAD and following acute myocardial infarction. Fujimoto et al. showed that in patients undergoing early coronary intervention for acute myocardial ischemia, low peak oxygen consumption <16.3 mL/kg/min and a high V_E_/VCO_2_ slope (>36.2) were predictive of future adverse events [50].

Eventually, De Assumpção et al. [77] examined the relationship between CPET-derived variables and long-term outcomes, focusing on the prognostic value of ventilatory efficiency (V_E_/VCO_2_ slope) and peak VO_2_, demonstrating that both V_E_/VCO_2_ slope and peak VO_2_ were strong predictors of adverse cardiovascular events, such as hospitalization and mortality.

In conclusion, CPET provides important additional information compared to traditional exercise testing in the context of known and suspected CAD.

### 3.6. Special Populations of Interest

Compared to traditional exercise stress testing, CPET offers a broader spectrum of additional information with value for specific subgroups of CAD patients.

Women. CPET has demonstrated greater diagnostic accuracy than traditional stress testing, more effectively differentiating between physiological and pathological responses. Women have been shown to have a high incidence of non-obstructive CAD (NOCAD), a condition involving ischemia due to microcirculatory disease, which is correlated with an increased risk of adverse events and is often undetectable by traditional exercise testing [78]. During CPET, these patients exhibited cardiac impairments such as altered chronotropic response and SV [79]. In addition, women show greater reproducibility in peak VO_2_ and oxygen pulse values compared to men in various studies [20].

Athletes. CPET has proven effective in identifying ischemic abnormalities in athletic cohorts with NOCAD, as indicated by a diminished O_2_-pulse slope, a lower DVO_2_/DWR ratio, and an augmented ΔHR-WR slope after the AT [29,39].

Heart failure. CPET is a cornerstone in evaluating and managing patients with heart failure [24,25,26,80]. Peak VO_2_ and oxygen pulse, derived from CPET, serve as robust prognostic markers, offering invaluable insights into the severity of myocardial impairment and guiding therapeutic strategies. In this context, CPET can distinguish changes in gas exchange parameters due to ischemia from those primarily caused by cardiac impairment.

Beta-Blocker Therapy. CPET remains informative for patients on beta-blocker therapy, which, while improving prognosis, does not significantly alter peak VO_2_ [24,81]. Studies have shown that cardioselective beta-blockers, such as bisoprolol, do not affect ventilatory responses, as evidenced by unchanged V_E_/VCO_2_ slopes and P_ET_CO_2_ levels [24].

ECG-inconclusive cases. CPET can provide valuable diagnostic information in cases where ECG findings are inconclusive. For example, in patients with left bundle branch block (LBBB), traditional exercise testing often fails to diagnose ischemia definitively, and dobutamine stress echocardiography is limited due to septal motion abnormalities. In these patients, an incongruent chronotropic or pressor response, as well as impaired increases in oxygen uptake, oxygen pulse, and the O_2_/WR ratio, suggests cardiac impairment and underlying ischemic heart disease [19,82].

Furthermore, CPET can help differentiate between cardiac, pulmonary, and muscular causes of exercise intolerance in patients with atypical symptoms [83].

In patients with ischemic heart disease, CPET is a valuable tool for assessing the response to anti-ischemic therapy. Improvements in myocardial perfusion and contractility following treatment with ACE inhibitors, ARBs, and beta-blockers should result in increased peak SV and peak VO_2_ [76]. CPET has also proven useful in the rehabilitation setting, particularly for post-MI patients and those with heart failure. The observed progressive increase in peak VO_2_ during rehabilitation indicates improved functional capacity and therapeutic efficacy [84].

### 3.7. Tailoring Treatment and Integration of CPET in the Current Management of CAD

The integration of CPET into CAD management presents a significant opportunity to refine treatment strategies, going beyond the limitations of traditional diagnostic approaches. CPET’s ability to evaluate the dynamic interaction between the cardiovascular, pulmonary, and muscular systems during exercise provides a multidimensional perspective on patient health, making it a valuable tool in personalized medicine, especially for those patients presenting with dyspnea as a primary symptom. Traditional diagnostic tests, such as the ECG stress test and imaging modalities like CCT, stress echocardiography, SPECT, and CMR, are well established in clinical guidelines. However, CPET may offer several pathophysiological advantages that could enhance CAD management.

Thus, one of the most compelling reasons to integrate CPET into clinical practice is its capacity to tailor treatment to the individual patients’ needs. Unlike traditional exercise tests, which focus primarily on electrocardiographic changes, CPET provides a comprehensive assessment of the body’s response to exercise, including measurements of VO_2_ max, ventilatory efficiency, and oxygen pulse. These parameters offer a detailed picture of the patient’s cardiorespiratory fitness and the efficiency of their cardiovascular system under stress, identifying early signs of ischemia or other cardiac dysfunctions that might not be apparent through other tests.

CPET’s utility extends beyond diagnostic precision. It can assist in tailoring exercise prescriptions, optimizing pharmacologic therapy, and determining the appropriate timing for interventions such as revascularization or advanced therapies for heart failure patients. For instance, in CAD patients, the AT is a critical marker that indicates when the heart can no longer meet the body’s oxygen demands through aerobic metabolism alone. This information is invaluable for structuring safe and effective cardiac rehabilitation programs, ensuring that exercise intensity is adjusted to the patient’s functional capacity, and avoiding the exacerbation of ischemic episodes.

CPET also has significant prognostic value. Parameters such as VO_2_ max and the V_E_/VCO_2_ slope have been strongly correlated with long-term outcomes in CAD patients. As presented, a lower VO_2_ max is associated with higher mortality rates, while an abnormal V_E_/VCO_2_ slope suggests poorer ventilatory efficiency and predicts adverse cardiovascular events. By incorporating CPET into routine clinical practice, clinicians can stratify patients more effectively according to their risk profiles, potentially leading to more targeted interventions and improved survival rates and quality of life for CAD patients.

Despite its advantages, integrating CPET into clinical guidelines for CAD management faces several challenges. One significant barrier is the relative novelty of CPET compared to more established tests like the ECG stress test, which has been a cornerstone of CAD diagnosis for decades. Although evidence supporting CPET is growing, the number of large-scale studies is still relatively small compared to those validating traditional exercise testing, leading to hesitancy among clinicians and guideline committees.

Another challenge is the specialized training required to administer and interpret CPET accurately. The complexity of the test demands a high level of expertise to ensure reliable results, potentially limiting its widespread adoption, particularly in resource-constrained settings where access to trained personnel may be limited. Additionally, the specific equipment and the time-intensive nature of the test may pose logistical challenges, further complicating its routine integration into clinical workflows.

From an economic standpoint, CPET presents a favorable cost/benefit ratio, particularly when compared to more expensive imaging modalities such as MRI stress tests, CCT, or SPECT. However, the initial costs associated with training and equipment, coupled with the longer duration of the test, must be considered when evaluating its overall impact on healthcare costs. Nonetheless, the long-term savings achieved through more precise diagnostics and tailored treatments could offset these initial investments, particularly in healthcare systems aiming to improve efficiency and patient outcomes.

From a recommendation standpoint, current clinical guidelines [4] emphasize the priority of functional tests in the diagnostic pathway for CAD, especially when anatomical testing alone is insufficient to inform treatment decisions. CPET could play a key role here, offering the benefits of traditional stress testing while providing additional insights into the pathophysiological impact of CAD. However, it is important to acknowledge that CPET has limitations, particularly in localizing ischemia and quantifying coronary artery obstruction—key factors in revascularization decisions [4]. As a result, while CPET is an excellent tool for assessing overall cardiovascular function, guiding therapeutic decisions and providing information on multiple systems during exercise that other tests cannot offer, it may need to be combined with imaging modalities like CCT or stress MRI to provide a comprehensive diagnostic approach, especially when revascularization is being considered.

In conclusion, although CPET is not yet fully integrated into clinical guidelines for CAD, its potential to enhance diagnostic accuracy, personalize treatment, and improve prognostic assessment is clear. To fully establish its role alongside traditional diagnostics, further large-scale studies are necessary. Additionally, standardizing training and testing protocols will be critical to overcoming barriers to wider adoption. As evidence grows, CPET could become a central component of a more personalized approach to managing CAD, offering a cost-effective and comprehensive tool for optimizing patient care across various clinical settings.

Table 2 highlights the differences between CPET, the ergometric test, and other functional tests recommended in clinical guidelines.

## 4. Conclusions

CPET is a powerful diagnostic and prognostic tool for the detection and the management of chronic coronary syndrome, further offering a comprehensive analysis of cardiovascular, pulmonary, and skeletal muscle responses during exercise. Its ability to capture subtle physiological data makes it more beneficial in populations where traditional exercise testing falls short, such as older adults, inconclusive ECGs, and athletes. CPET may provide detailed insights into cardiopulmonary function, often missed by standard assessments, enhancing both diagnostic precision and the ability to predict adverse cardiovascular events through parameters like VO_2_ peak and V_E_/VCO_2_ slope.

This review advocates for the broader integration of CPET into clinical practice. By improving diagnostic accuracy, refining risk stratification, and guiding personalized therapeutic decisions, CPET has the potential to significantly enhance CAD management. Future research should focus on standardizing CPET protocols and exploring its use across a broader range of clinical settings to fully harness its potential. This integration will be critical for advancing tailored treatment plans and improving long-term outcomes in CAD management.

## Figures and Tables

**Table 2:** Advantages of CPET vs. traditional exercise tests and functional tests recommended in current clinical guidelines.

Aspect	CPET [5]	Traditional Exercise Test	Imaging-Based Functional Tests: CMR, SPECT, PET [85]
Sensitivity	86–87% [1,6]	45–70% [15,19]	86% (CMR), 83% (SPECT), and 85% (PET) [85]
Specificity	74–98% [1,6]	70–80% [15]	83% (CMR), 77% (SPECT), and 86% (PET) [85]
Influence of medications	Low	High (e.g., beta-blockers)	Variable
Prognostic value	Strong (e.g., V_E_/VCO_2_, VO_2_ max)	Moderate (Duke Treadmill Score)	Strong (perfusion defects, wall motion)
Special population suitability	High (athletes, women, heart failure)	Low	Moderate (depending on imaging modality)
Exercise capacity assessment	Detailed	Basic	Limited to imaging stress capability
Risk stratification	Comprehensive	Basic (based on exercise tolerance)	Advanced (perfusion, wall motion, viability)

## Supplementary Materials

The following supporting information can be downloaded at: https://www.mdpi.com/article/10.3390/jcdd11110357/s1, Table S1 contains a resume of the main presented studies throughout the review [86,87,88].
