# Supplementary material for: The Complementary Role of Cardiopulmonary Exercise Testing in Coronary Artery Disease: From Early Diagnosis to Tailored Management
Source: J Cardiovasc Dev Dis. 2024 Nov 5;11(11):357. doi: 10.3390/jcdd11110357 (PMC11594985; doi:10.3390/jcdd11110357)
Supplement: Supplementary file 1 [file jcdd-11-00357-s001.zip › Supplementary Table S1.pdf]

Table S1. Presentation of the main discussed studies.

Authors	Variables	N.Patients	Results	Impact and limitations
**CPET vs stress ECG**				
Belardinelli et al (2003) [5]	O_2 pulse, $\Delta VO_2/\Delta WR$ slope	202	The study demonstrated significantly higher sensitivity (87%) and specificity (74%) in identifying exercise-induced myocardial ischemia (EIMI) with CPET compared to stress ECG (46% sensitivity, 66% specificity), with notable O_2 pulse flattening duration and $\Delta VO_2/\Delta WR$ slope predictive of EIMI.	This study was pivotal in highlighting CPET's ability to detect ischemia, particularly in patients with a higher global ischemic burden.
Bussotti et al (2006) [30]	Peak VO_2, $\Delta VO_2/\Delta WR$ slope after the AT	48	Patients with epicardial disease exhibited significantly lower peak VO_2 (68%) and a flattening of the $\Delta VO_2/\Delta WR$ slope post-AT, compared to those without epicardial disease and to healthy controls.	The study suggests that individuals with ST depression but without significant epicardial disease may experience microvascular ischemia, indicating the need for further evaluation, including assessments of endothelial dysfunction and coronary flow reserve (CFR).
Chaudhry et al (2017) [20]	ΔHR-WR slope	208	The study found that the novel ΔHR-WR slope parameter significantly improved CPET's sensitivity in detecting atherosclerotic heart disease, with specificity similar to that of stress ECG.	This was the first study to demonstrate that abrupt HR acceleration after the VAT could be used diagnostically to identify EIMI, indicating that CPET could detect microvascular ischemia independently of the degree of luminal narrowing.

CPET vs coronary angiography				
**Authors**	**Variables**	**N.Patients**	**Results**	**Impact and limitations**
Tajima et al (2009) [37]	$\Delta VO_2/\Delta WR$ slope	37	Patients with CAD showed a significant decrease in the $\Delta VO_2/\Delta WR$ slope above VAT and IT, along with delayed VO_2 recovery kinetics compared to those without CAD.	This study emphasized the value of CPET in assessing cardiac output dynamics and provided a clear link between CPET and traditional imaging modalities such as coronary angiography.
Belardinelli et al (2014) [8]	Peak VO_2, $VO_{2\,max}$	1265	CPET significantly improved sensitivity (from 48% with stress ECG to 88% with CPET), and specificity (from 55% to 98%) for detecting myocardial ischemia. Patients with both peak VO_2 > 91% of predicted VO_2 max and absence of VO2-related signs of myocardial ischemia had no evidence of O-CAD in 100% of cases.	These findings advanced the field by demonstrating CPET's superiority over traditional stress ECG for diagnosing myocardial ischemia and predicting the absence of O-CAD, suggesting that CPET should be considered a first-line diagnostic tool in clinical practice.
Mazaheri et al (2016) [34]	V_E/VCO_2 slope, O_2 pulse	31	A ventilatory equivalent for carbon dioxide (V_E/VCO_2) greater than 35 predicted a positive coronary angiogram, indicating the presence of CAD. The O_2 pulse plateau duration was similar between the obstructive CAD (OCAD) and non-OCAD groups.	The studyhighlighted limitations in the statistical analysis, particularly regarding O_2 pulse plateau duration, which was similar across the OCAD and non-OCAD groups. Despite the reduced peak VO_2 observed in the OCAD group, this results was not statistically significant. This work emphasized the potential of V_E/VCO_2 as a predictive marker for CAD while underscoring the need for rigorous statistical methods in CPET research.
Popovic et al. (2017) [14]	ΔVCO_2, ΔVO_2	40	The study found that changes in ΔVCO_2 and ΔVO_2 recovery-to-peak ratios during CPET significantly distinguished patients with one- or two-vessel coronary artery stenosis from those with three-vessel stenosis.	The study emphasized that a higher global ischemic burden appears to be linked to impaired pulmonary gas exchange during recovery, suggesting that CPET during the recovery phase could be a valuable predictor of CAD severity.

Yoshida et al. (2017) [32]	HR	40	The study demonstrates that HR augmentation after the ischemic threshold is a key driver of cardiac output and peak VO_2. It also highlighted that beta-blockers may induce exercise intolerance by blunting the compensatory HR response after the ischemic threshold.	The research provided critical insights into the dependence of cardiac output on HR and SV during exercise, especially in the context of pharmacological interventions.
Niu et al. (2020) [41]	Duration exercise, peak VO_2, HRR, V_E/VCO_2 slope	184	The study identified four CPET variables as predictive of cardiovascular events post-PCI. Reduced peak CO and chronotropic incompetence were associated with poor prognosis in CAD patients.	The study highlighted that developing pulmonary congestion during exercise, due to impaired left ventricular function, is a sign of heart failure, which CPET can effectively identify.
Li N. et al. (2022) [6]	peak VO_2, AT, peak $V\ O_{2/Kg}$, peak O_2 pulse, maximum exercise load, maximum metabolic equivalent, and exercise time	155	Results showed that all CPET parameters were significantly lower in the CAD group and negatively correlated with coronary artery stenosis severity, as measured by the Gensini score.	The study demonstrated that CPET, as a non-invasive and comprehensive assessment tool, has high sensitivity (86.4%) and specificity (98.5%) when combining multiple indices, highlighting its potential for diagnosing CAD and assessing coronary artery stenosis severity.
Li S. et al. [31] (2022)	peak VO_2, HR	715	The study found that mean peak VO_2 decreased proportionally with an increasing OCAD burden, showing a clear gradient from healthy individuals to those with severe OCAD. Peak SV and peak HR similarly declined as atherosclerosis burden increased.	The study emphasized the association between large-vessel atherosclerosis and progressively impaired cardiac output and chronotropic response at CPET, suggesting the need for aggressive follow-up in patients with non-OCAD who showed lower peak VO_2 than healthy volunteers.
Liu et al. (2022) [7]	peak VO_2, VAT	280	The study demonstrated a strong inverse association between peak VO_2, VAT, and OCAD severity, as measured by quantitative flow ratio (QFR), the number of lesions > 50%, and the Gensini score.	The findings suggested that incorporating CRF information into clinical models could significantly enhance risk estimation accuracy, reinforcing CPET's value as a non-invasive tool for stratifying OCAD severity.

CPET vs coronary angiography or coronary CT angiogram				
**Authors**	**Variables**	**N.Patients**	**Results**	**Impact and limitations**
Petek et al. (2021) [35]	O_2 pulse, HR-WR	104	The findings indicated that an O_2 pulse plateau alone was not a useful predictor of OCAD in this population, suggesting that the O_2 pulse parameter should be integrated with other CPET metrics rather than used in isolation. The study emphasized that O_2 pulse trajectories must become abnormal shortly after the AT and be accompanied by heart rate-work rate (HR-WR) acceleration to be considered pathological. A gradual plateau without HR acceleration was regarded as normal, making it challenging to differentiate normal from abnormal trajectories in many cases.	This study contributed to understanding that CPET, while valuable, requires careful interpretation of O_2 pulse data, especially in populations with potential microvascular dysfunction.
**CPET, myocardial perfusion scintigraphy (MPS), and coronary CT angiogram**				
Van de Sande et al. (2019) [39]	ΔHR-WR slope, O_2 pulse	156	The findings showed that athletes with abnormal stress ECGs demonstrated an attenuated O_2 pulse slope, a decreased $\Delta VO_2/\Delta WR$ ratio, and an increased heart rate-work rate (ΔHR-WR) slope post-AT, consistent with microvascular ischemia	This was the first study to validate the ΔHR-WR slope as a marker for exercise-induced myocardial ischemia (EIMI) in an asymptomatic athletic population, suggesting that this methodology is very sensitive and could serve as an early marker for microvascular dysfunction.

CPET vs single photon emission computed tomography (SPECT)				
**Authors**	**Variables**	**N.Patients**	**Results**	**Impact and limitations**
Uliari et al (2016) [38]	$\Delta VO_2/\Delta WR$ slope	29	The study found a significant decrease (by an average of 44.6%) in the $\Delta VO_2/\Delta WR$ slope above VAT in 23 of the 26 patients in whom VAT could be determined, corroborating previous observations in cardiac patients and indicating an attenuation in oxygen uptake at submaximal exercise intensities.	This study was pivotal in demonstrating that a decrease in $\Delta VO_2/\Delta WR$ slope after AT could be observed during submaximal exercise.
Munhoz et al (2007) [33]	Peak O_2 pulse, peak VO_2	87	Patients with extensive ischemia had significantly reduced peak O_2 pulse and peak VO_2.	This research showed that reduced peak SV and CO were linked with high ischemic burdens. However, the diagnostic value was limited by the lack of detailed analysis of the O_2-pulse trajectory and the inherent limitations of nuclear stress testing, particularly its lower sensitivity for detecting microvascular and balanced ischemia.

CPET, cardiac MRI and transthoracic echocardiogram				
**Authors**	**Variables**	**N.Patients**	**Results**	**Impact and limitations**
Gulsin et al. (2020) [86]	Peak VO_2	325	The study found that subjects with type 2 diabetes (T2D) had increased concentric left ventricular remodeling, reduced myocardial perfusion reserve (MPR), and markedly lower aerobic exercise capacity (peak VO_2 18.0 vs. 27.8 mL/kg/min) compared to controls. Only MPR and left ventricular diastolic filling pressure were independently associated with peak VO_2 in subjects with T2D, indicating that microvascular dysfunction and diastolic dysfunction are key drivers of reduced exercise capacity in this population.	This study underscores the importance of assessing subclinical cardiac dysfunction in high-risk populations using CPET.

CPET alone				
**Authors**	**Variables**	**N.Patients**	**Results**	**Impact and limitations**
Almeida et al. (2022) [87]	O_2 pulse	824	The findings revealed that an early flattening of the O_2 pulse was associated with cardiovascular risk factors, as an early marker of subclinical cardiac dysfunction in an otherwise asymptomatic population.	This study underscored the potential of CPET to detect early signs of cardiovascular disease in populations that may otherwise be overlooked using standard risk assessments.
Smith et al. (2022) [88]	Peak VO_2	967	The findings showed that nearly two-thirds of participants had subclinical cardiac dysfunction despite normal predicted CRF (median peak VO_2 = 102%). Subclinical dysfunction was significantly associated with advanced age, obesity, diastolic hypertension, high triglycerides, low high-density lipoprotein (HDL) cholesterol, and reduced peak VO_2, with the strongest associations observed for obesity and diastolic hypertension.	This study highlights that subclinical cardiac dysfunction is prevalent even in asymptomatic, at-risk populations and is associated with reduced cardiorespiratory fitness, suggesting a poorer prognosis.
**CPET with stress echocardiography (CPET-SE)**				
Smarz et al. (2021) [42]	Exercise capacity	81	The study found that exercise capacity in patients with left ventricular ejection fraction (LVEF) >40% was related to peak HR and peripheral oxygen extraction, but not peak SV.	This research highlights the importance of integrating CPET with other modalities to fully understand exercise capacity limitations in post-AMI patients.
De Assumpção et al. (2021) [77]	V_E/VCO_2 slope, peak VO_2	92	Results demonstrated that both higher V_E/VCO_2 slope and lower peak VO_2 were strong predictors of adverse cardiovascular events, such as hospitalization and mortality.	This study contributes to the growing evidence supporting CPET as a prognostic tool in managing stable CAD, particularly for predicting long-term outcomes and guiding therapeutic strategies.
Ganesananthan et al. (2022) [36]	O_2 pulse	195	The findings showed that patients exhibiting an O_2 pulse plateau during CPET had higher ischemic dobutamine stress echocardiography (DSE) scores and lower fractional flow reserve (FFR) compared to those without an O_2 pulse plateau.	This study emphasizesthe importance of specific CPET parameters in evaluating ischemic heart disease, suggesting that CPET could play a crucial role in selecting patients likely to benefit from PCI.

CPET and transthoracic Doppler echocardiography (TTDE) with dipyridamole-induced vasodilation				
**Authors**	**Variables**	**N.Patients**	**Results**	**Impact and limitations**
Bechsgaard et al (2019) [40]	VO_2, HRR	126	The study found that coronary microvascular dysfunction (CMD) was present in 27% of the symptomatic women and 19% of controls. Notably, peak VO_2 was significantly reduced in patients with CMD (17.3 vs. 27.3 ml/kg/min), independent of other cardiovascular risk factors. It was also associated with diminished heart rate reserve and blunted heart rate recovery.	This study aimed to determine whether exercise capacity was reduced in women with CMD compared to sex-matched controls.
